# CD106 Identifies a Subpopulation of Mesenchymal Stem Cells with Unique Immunomodulatory Properties

**DOI:** 10.1371/journal.pone.0059354

**Published:** 2013-03-12

**Authors:** Zhou Xin Yang, Zhi-Bo Han, Yue Ru Ji, You Wei Wang, Lu Liang, Ying Chi, Shao Guang Yang, Li Na Li, Wei Feng Luo, Jian Ping Li, Dan Dan Chen, Wen Jing Du, Xiao Cang Cao, Guang Sheng Zhuo, Tao Wang, Zhong Chao Han

**Affiliations:** 1 The State Key Laboratory of Experimental Hematology, Institute of Hematology and Hospital of Blood Diseases, Chinese Academy of Medical Sciences and Peking Union of Medical College, Tianjin, China; 2 National Engineering Research Center of Cell Products, Tianjin, China; 3 Beijing Institute of Health and Stem Cells, Beijing, China; 4 School of Medicine, Jinan University, Guangzhou, China; 5 Department of Gastroenterology and Hepatology, Tianjin Medical University General Hospital, Tianjin Medical University, Tianjin, China; Children's Hospital Boston, United States of America

## Abstract

Mesenchymal stem cells (MSCs) reside in almost all of the body tissues, where they undergo self-renewal and multi-lineage differentiation. MSCs derived from different tissues share many similarities but also show some differences in term of biological properties. We aim to search for significant differences among various sources of MSCs and to explore their implications in physiopathology and clinical translation. We compared the phenotype and biological properties among different MSCs isolated from human term placental chorionic villi (CV), umbilical cord (UC), adult bone marrow (BM) and adipose (AD). We found that CD106 (VCAM-1) was expressed highest on the CV-MSCs, moderately on BM-MSCs, lightly on UC-MSCs and absent on AD-MSCs. CV-MSCs also showed unique immune-associated gene expression and immunomodulation. We thus separated CD106^+^cells and CD106^−^cells from CV-MSCs and compared their biological activities. Both two subpopulations were capable of osteogenic and adipogenic differentiation while CD106^+^CV-MSCs were more effective to modulate T helper subsets but possessed decreased colony formation capacity. In addition, CD106^+^CV-MSCs expressed more cytokines than CD106^−^CV-MSCs. These data demonstrate that CD106 identifies a subpopulation of CV-MSCs with unique immunoregulatory activity and reveal a previously unrecognized mechanism underlying immunomodulation of MSCs.

## Introduction

In recent years, mesenchymal stem cells have attracted significant attention from basic and clinical investigators for their usefulness in the treatment of immune disorders, such as graft-versus-host disease (GVHD) and autoimmune diseases [1], [2]. MSCs constitute a specialized tissue microenvironment or niche, where they undergo self-renewal and multi-lineage differentiation [3]. MSCs were first isolated from bone marrow [4], and subsequently found in nearly every tissue attempted so far, including adipose [5], and several birth associated perinatal tissues including placenta [6], umbilical cord [7] and cord blood [8].

A number of studies have shown that MSCs derived from different tissues share many similarities but also exhibit some differences. Using identical culture conditions, major differences were observable in the frequencies, proliferation and differentiation potentials as well as biological functions [7], [9]. Placental and umbilical cord derived MSCs are more primitive because they share more common genes with embryonic stem cells (ESCs) [10]. UC-MSCs express genes enriched in vascular endothelial growth factor and PI3K-NFκB canonical pathways, whereas BM-MSCs express genes involved in antigen presentation and chemokine/cytokine pathways. UC-MSCs thus constitute a promising option for angiogenesis, whereas BM-MSCs for bone remodeling [11]. Furthermore, it has been found that the migration of placental and BM-MSCs was found to be 5.9 and 3.2 folds higher than that of UC-MSCs, respectively [12]. BM-MSCs show a stronger stimulating effect on megakaryocyte progenitor expansion than those from UC-MSCs [13] and display a better chondrogenic differentiation compared with other sources of MSCs [14]. In addition, the cytokine profiling is different based on the sources of MSCs [15]. MSCs from different tissues display different potential in proliferation, differentiation, immunomodulation and hematopoiesis supportive abilities, and these differences indicate a demand for effective preparation protocols tailored to each type of MSCs against different diseases [16].

One of the important biological functions of MSCs is the immunomodulation. MSCs can alter the function of T cells, B cells, dendritic cells and NK cells, and thereby exhibit potent immunosuppressive activity [17]. Generally, MSCs display their immunomodulatory activities through direct cell-cell contact and/or secretion of soluble factors [17]. Cell – cell adhesion mediated by CD106 is known to be critical for T cell activation and leukocyte recruitment to the inflammation site and, therefore, plays an important role in evoking effective immune responses. CD106 is also reported as one of components of neural stem cells niche [18]. Moreover, CD106 is critical for MSCs-mediated immunosuppression [19] and for the binding of hematopoietic progenitor cells [20].

In the present study, we report a positive correlation between CD106 expression and immunosuppressive effect of CV-MSCs. We also show that TNF-α and IL-1β are required for CD106^+^MSCs expansion. Our data suggest that CD106 could be used as a biomarker for a subpopulation of CV-MSCs with unique immunosuppressive activity.

## Materials and Methods

### Generation of human BM-MSCs, AD-MSCs, UC-MSCs and CV-MSCs

This study was approved by the Institutional Review Board of Chinese Academy of Medical Sciences and Peking Union Medical College. Bone marrow, adipose tissues, term placenta, umbilical cords, peripheral blood and cord blood were obtained from donors with written informed consent.

The isolation and expansion of MSCs were performed as described previously [7]. The basic culture medium for isolation of MSCs was the complete DF-12 medium (Gibco) containing 10% fetal calf serum (FCS) (HyClone), 2 mM glutamine, 100U/ml penicillin-streptomycin, and 10 ng/ml epidermal growth factor (EGF; Peprotech). Samples from 3 donors each were used to generate human BM-MSCs, AD-MSCs, UC-MSCs and CV-MSCs.

### Flow cytometric analysis

Phenotype of MSCs was analyzed using the following antibodies: FITC- conjugated-CD19, CD31 and CD34; PE-conjugated-CD11b, CD44, CD45, CD54, CD73, CD90, CD105, CD106 and HLA-DR. Non-specific isotype-matched antibodies served as controls. For Tregs, CD4^+^T cells were labeled with PE-conjugated CD4, FITC- conjugated CD25, and then labeled PE-Cy5-conjugated Foxp3 by using Foxp3 Staining Buffer Set (eBioscience). All of the antibodies were purchased from BD Pharmingen. Cells were analyzed by flow cytometry in a FACS Calibur, using the CellQuest software (Becton Dickinson).

### Separation of CD106^+^CV-MSCs and CD106^−^CV-MSCs

Subpopulations of MSCs were separated using EasySep® PE Positive Selection Kit (StemCell Technologies). MSCs were detached by 0.025% trypsin and labeled with PE-anti CD106 antibody, and CD106^+^ and CD106^−^ cells were separated according to manufacturer's instructions. In some cases, flow sorting was used. MSCs were labeled with PE-anti CD106 antibody, the positive and negative cells were separated using BD Influx (Becton Dickinson). For both of the methods, the purity of isolated cells was more than 90%.

### Osteogenic and adipogenic differentiation

Osteogenic and adipogenic differentiation was carried out as described previously [7]. Briefly, cells were plated in 24-well plates at a density of 3000 cells/cm^2^. The medium was changed with specific induction medium 24 hours later. For osteogenic induction, STEMPRO Osteogenesis Differentiation Kit (GIBCO) was used. For adipogenic induction, medium consisted of DMEM supplemented with 10% FBS, 1 µmol/L dexamethasone, 5 µg/mL insulin, 0.5 mmol/L isobutylmethylxanthine (IBMX), and 60 µmol/L indomethacin was used. Reagents for adipogenic induction were purchased from Sigma. After 3 weeks of induction, the cells were stained using alizarin red S or oil red O solution.

### Colony forming unit-fibroblast assay

For fibroblastic-like colony formation assay, 3 cells/cm^2^ of CD106^+^ or CD106^−^ CV-MSCs were seeded in T75 culture flask. The cell media were changed every 7 days. The culture was ended at day 14, and the adherent cells were washed twice with phosphate-buffered saline, and stained with 0.2% crystal violet in 80% methanol for 20 min at room temperature. Then the cells were washed again and the colonies were counted.

### Isolation of human peripheral blood mononuclear cells (hPBMCs) and human cord blood mononuclear cells (hCBMCs) CD4^+^T cells

Human PBMCs and CBMCs were isolated by Ficoll-Paque (Axis-Shield) density gradient centrifugation from blood of health volunteer donors. CD4^+^T cells were purified using relevant magnetic MicroBead kits (Miltenyi Biotec) according to the manufacturer's instructions. The purity of CD4^+^T cells was more than 95%.

### Co-culture of immune cells and MSCs

Cells were cultured in complete 1640 medium (Gibco) containing 10% fetal bovine serum (FBS) (FBS- NZ ORIGIN), 2 mM glutamine, 100 U/ml penicillin and streptomycin, 0.1 mM nonessential amino acids, 1 mM sodium pyruvate. MSCs (30 Gy irradiated) were plated in 96-well flat-bottom plate and allowed to adhere for 18 h at 37°C, and then 10^5^ PBMCs or CD4^+^T cells were added in. Human PBMCs were stimulated by phytohemagglutinin (PHA, Sigma, 10 μg/ml) and CD4^+^T cells were stimulated by PHA (10 μg/ml) and IL-2 (peprotech, 10 ng/ml) for 72 hours. For the regulatory T cells (Tregs), CD4^+^T cells were stimulated with IL-2 (10 ng/ml) for 5 days. Cells were isolated and labeled with CD4, CD25 and Foxp3.

### Affymetric Genechip assay

2×10^6^ of CD106^+^ and CD106^−^ CV-MSCs was collected and resuspended in Trizol (Invitrogen). The samples were stored in −80°C for genechip assay. The Microarray was done by CapitalBio Corporation. Data was analyzed using mas3.0 molecule annotation system.

### RNA isolation, reverse transcription and real time PCR

Total RNA was extracted by E.Z.N.A. Total RNA Kit I (OMEGA), and cDNA synthesis with MLV RT kit (Invitrogen) for 50 min at 37°C in the presence of oligo-dT primer. Real-time polymerase chain reaction analyses were performed by Platinum® SYBR® Green qPCR SuperMix-UDG w?ROX (invitrogen) on an Applied Biosystems 7300 Real-Time PCR System. Primers used were listed in Table 1.

**Table 1:** Primers for real time reverse transcription – polymerase chain reaction.

Human β-actin forward:	5′-CAGAGCAAGAGAGGCATCC-3′
Human β-actin reverse:	5′-CTGGGGTGTTGAAGGTCTC-3′
Human VCAM-1 forward:	5′-GGCAGAGTACGCAAACACTT-3′
Human VCAM-1 reverse:	5′-GGCTGTAGCTCCCCGTTAG-3′
Human COX-2 forward:	5′-ACTCTGGCTAGACAGCGTAA-3′
Human COX-2 reverse:	5′-ACCGTAGATGCTCAGGGAC-3′
Human IDO1 forward:	5′-AGACTGCTGGTGGAGGACATG-3′
Human IDO1 reverse:	5′-AAAGGACAAACTCACGGACTG-3′
Human TGF-β1 forward:	5′-ACTGCAAGTGGACATCAACG-3′
Human TGF-β1 reverse:	5′-TGCGGAAGTCAATGTACAGC-3′
Human IL-1α forward:	5′-CGCCAATGACTCAGAGGAAGA-3′
Human IL-1α reverse:	5′-GCAGCAGCCGTGAGGTACT-3′
Human IL-1β forward:	5′-AGCTACGAATCTCCGACCAC-3′
Human IL-1β reverse:	5′-CGTTATCCCATGTGTCGAAGAA-3′
Human IL-6 forward:	5′-CCACACAGACAGCCACTCAC-3′
Human IL-6 reverse:	5′-CCAGATTGGAAGCATCCATC-3′
Human IL-8 forward:	5′-TTGGCAGCCTTCCTGATTT-3′
Human IL-8 reverse:	5′-TCAAAAACTTCTCCACAACCC-3′
Human IFN-γ forward:	5′-GAGTGTGGAGACCATCAAGGAAG-3′
Human IFN-γ reverse:	5′-TGAGTTCATGTATTGCTTTG-3′
Human TNF-α forward:	5′-CGAGTGACAAGCCTGTAGC-3′
Human TNF-α reverse:	5′-GGTGTGGGTGAGGAGCACAT -3′
Human T-bet forward:	5′-GCTGTCACCACTGGAAGGAT-3′
Human T-bet reverse:	5′-TTGGTGTGGACTGAGATTGC-3′
Human HLA-G forward:	5′- GAGGAGACACGGAACACCAAG-3′
Human HLA-G reverse:	5′- GTCGCAGCCAATCATCCACT-3′

### In vitro stimulation of MSCs

10^5^ cells/ml MSCs were seeded in to a 6 well plate in 2 ml for 24 hours, and then treated with IFN-γ (30 ng/ml), IL-1β (10 ng/ml) or TNF-α (30 ng/ml) for 24 hours. All of the pro-inflammatory cytokines were purchased from peprotech.

### ELISA

Cell-free supernatants were collected and kept in refrigerator at −80°C. PGE_2_ ELISA kit was purchased from Cayman Chemicals. IFN-γ was tested using the kit from ebioscience. All of the ELISA assay kits were used following the supplier's instruction.

### Statistical analysis

The data were analyzed for statistic significance using the GraphPad Prism software. Data are presented as mean ± SEM. Student's unpaired t-test and ANOVA with bonferroni post hoc test were used to determine significance, p<0.05 was considered to be statistically significant.

## Results

### Placental chorionic villi MSCs highly express CD106

**Figure 1:**
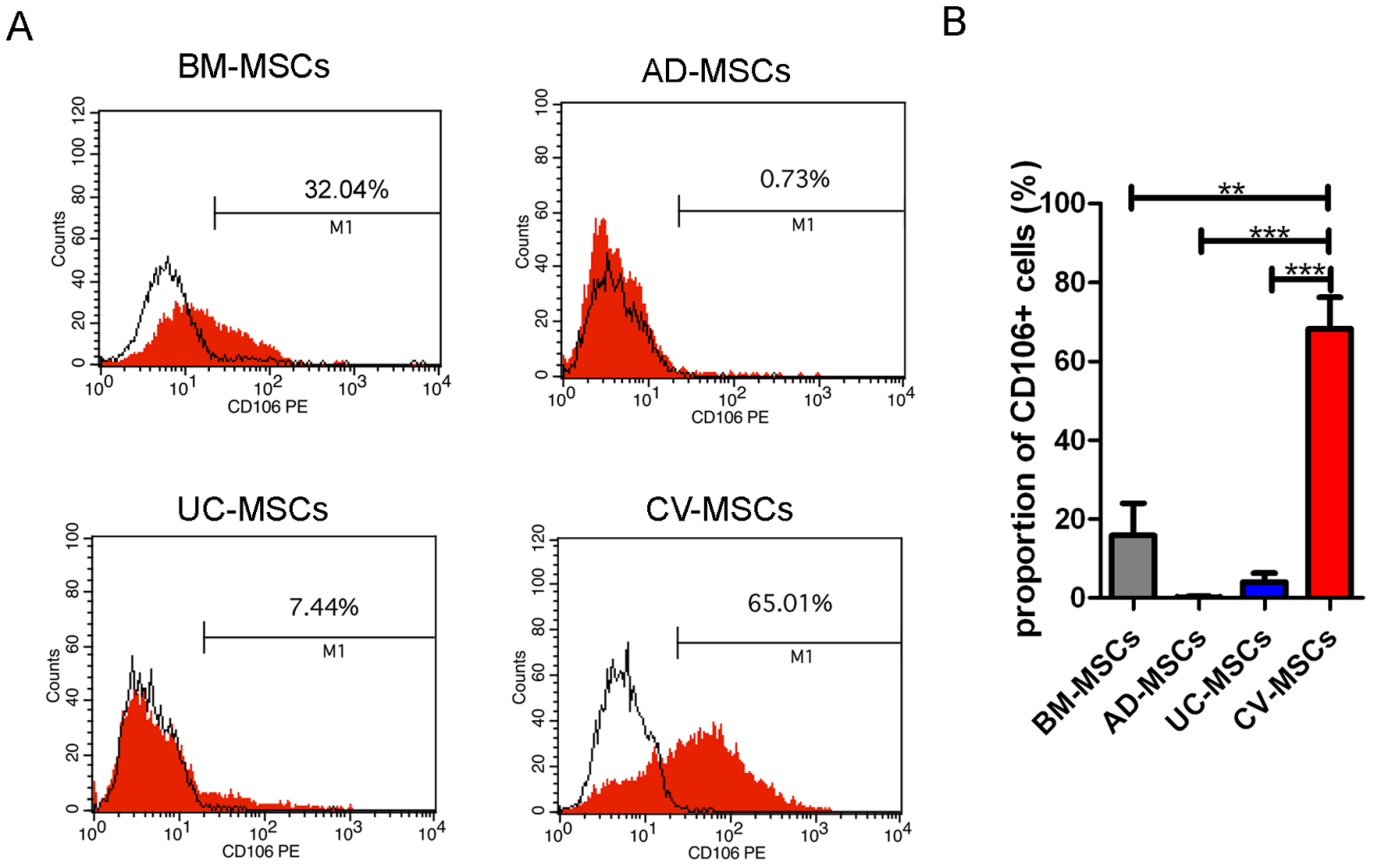
Comparison of CD106 expression in BM-MSCs, AD-MSCs, UC-MSCs and CV-MSCs. Cells of passage 3 were used. (**A**) FACS profiles of typical MSCs from different origins. (**B**) Statistic result of CD106 expression in different MSCs. Each type of MSCs was tested for 3 donors. (**p<0.01; ***p<0.001).

To determine most significant differences among MSCs of different origins, we first compared the phenotypes of MSCs isolated from human term placental CV, UC, adult BM and AD using identical methods. Each type of MSCs was tested for 3 donors. A series of cell markers were examined at passage 3 of in vitro cultivation, including CD11b, CD19, CD29, CD31, CD34, CD44, CD45, CD54, CD73, CD90, CD105, CD106, HLA-DR, Nestin and SOX2. All these four types of MSCs highly expressed embryonic stem cell markers Nestin and Sox2, as well as mesenchymal markers including CD29, CD44, CD73, CD90 and CD105 (Table 2). The most significant difference of their phenotypes was the CD106 expression, which was expressed highly on the CV-MSCs (68.26±7.970%), moderately on BM-MSCs (15.85±8.208%), lightly on UC-MSCs (4.067±2.197%) and absent on AD-MSCs (Figure 1A, B).

**Table 2:** Phenotype of BM-MSCs, AD-MSCs, UC-MSCs and CV-MSCs.

markers	BM-MSCs	AD-MSCs	UC-MSCs	CV-MSCs
CD11b	−	−	−	−
CD19	−	−	−	−
CD29	+++++	+++++	+++++	+++++
CD31	−	−	−	−
CD34	−	−	−	−
CD44	+++++	+++++	+++++	+++++
CD45	−	−	−	−
CD54	++	++++	+++	++++
CD73	+++++	+++++	+++++	+++++
CD90	+++++	+++++	+++++	+++++
CD105	+++++	+++++	+++++	+++++
CD106	+ or ++	−	+	++++
HLA-DR	−	−	−	−
Nestin	++++	+++++	+++++	+++++
SOX2	++	+++	+++++	++++

The markers of BM-MSCs, AD-MSCs, UC-MSCs and CV-MSCs were analyzed by flow cytometry (n = 3). – negative, +∼++++ positive, + 1–20%, ++ 20–40%, +++ 40–60%, ++++ 60–80%, +++++>80%.

### Gene expression pattern of placental chorionic villi MSCs is unique

**Figure 2:**
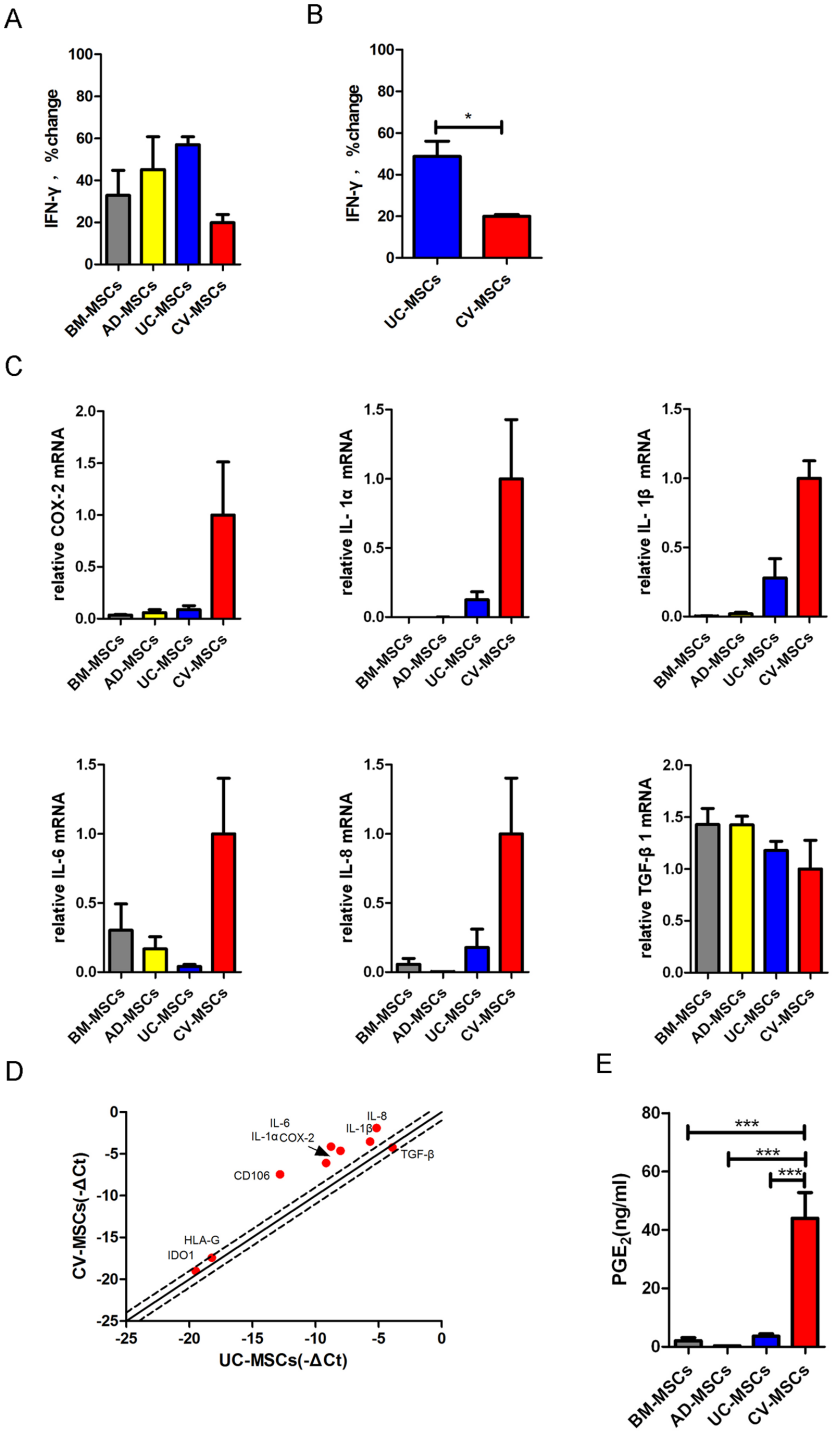
Comparison of different gene expression in BM-MSCs, AD-MSCs, UC-MSCs and CV-MSCs. (**A**) BM-MSCs, AD-MSCs, UC-MSCs and CV-MSCs were co-cultured with PHA (10 μg/ml) stimulated PBMCs for 72 hours. 10^5^ PBMCs and 2×10^4^ MSCs were added to a well of 96 well plates. IFN-γ production of activated-PBMCs was evaluated by ELISA. Percentage change of IFN-γ was gained by direct comparison to the control (activated PBMCs alone). (**B**) UC-MSCs and CV-MSCs from the same individuals were co-cultured with PHA stimulated PBMCs for 72 hours. A corresponding set of MSCs was tested. IFN-γ concentration in culture medium was measured by ELISA. Percentage change of IFN-γ was gained by direct comparison to the control (activated PBMCs alone). The results are presented as mean±SEM of single experiment, and the experiment have been performed at least in triplicate (*p<0.05), 3 donor samples were used. (**C**) Relative mRNA expression of COX-2, IL-1α, IL-1β, IL-6, IL-8 and TGF-β1 in BM-MSCs, AD-MSCs, UC-MSCs and CV-MSCs. Cells of passages 3 from 3 donors were used, respectively. (**D**) RT-PCR array results of immune related genes in UC-MSCs and CV-MSCs (from the same individual). –ΔCt (cycle threshold) were compared and shown in a scatter plot. (**E**) PGE_2_ concentration in culture medium of BM-MSCs, AD-MSCs, UC-MSCs and CV-MSCs after 72 hours culturing. The original seeding number is 10^5^/well of 6 well plates. Supernatant were obtained 72 hours later. Cells of passages 3 were used (from 3 donors) (***p<0.001).

To determine if different origins of MSCs affect Th1 specific cytokine IFN-γ expression, we then compared levels of IFN-γ using ELISA. PBMCs secreted IFN-γ upon stimulation of PHA (10 μg/ml). All of these MSCs suppressed the expression of IFN-γ in hPBMCs in the presence of PHA (Figure 2A). To exclude the possibility that the differences among MSCs were due to individual differences, we tested the UC-MSCs and CV-MSCs obtained from the same fetus origin. Interestingly, CV-MSCs (19.99±0.8328% compared with PHA-actived hPBMCs) showed a significantly increased inhibitory effect on IFN-γ production of the hPBMCs compared to UC-MSCs (48.74±7.323% compared with PHA-actived hPBMCs) (Figure 2B), indicating that placenta CV contain a valuable source of MSCs with increased immunomodulatory activity.

To examine the mRNA expression of several immune factors in the four sources of MSCs, realtime PCR was performed. It is interesting to note that CV-MSCs expressed highest level of mRNA for COX-2 (Cyclooxygenase-2, inducible synthase for PGE_2_), IL-1α, IL-1β, IL-6 and IL-8. However, the expression of TGF-β1 was similar in each type of MSCs (Figure 2C, D). The expression of IDO1 mRNA was very low in all sources unless treated by pro-inflammatory cytokines like IFN-γ. Importantly, CV-MSCs showed strongest ability to secrete PGE_2_ among the four sources of MSCs (44.00±8.814 ng/ml) (Figure 2E).

### CD106^+^CV-MSCs differ from CD106^−^CV-MSCs in their colony formation and immunosuppressive capacities

**Figure 3:**
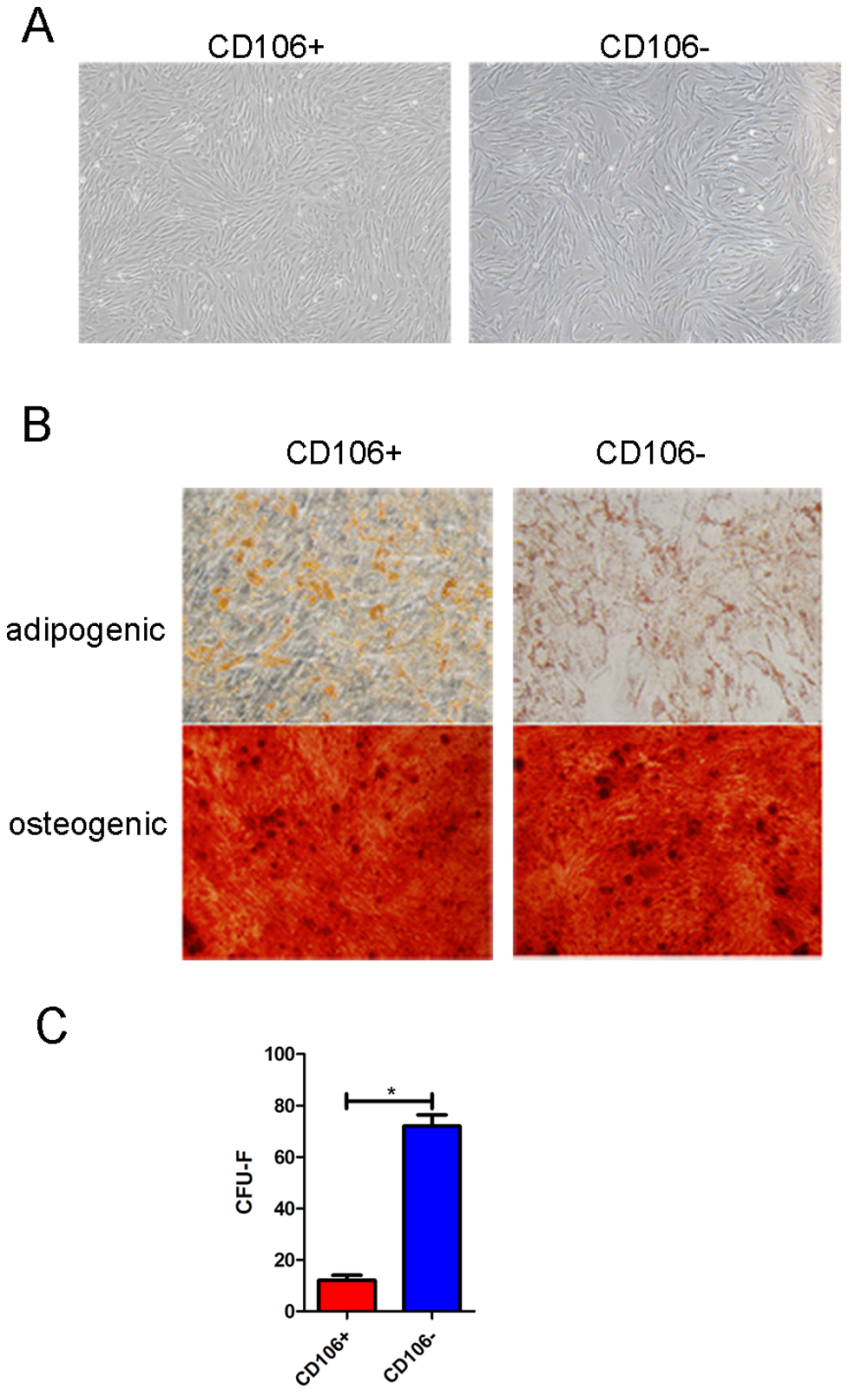
Isolation of CD106^+^cells and CD106^−^cells from CV-MSCs. Image for morphology and differentiation of MSCs were taken by OLYMPUS DP72. (**A**) Morphology of CD106^+^ and CD106^−^ cells from CV-MSCs. (**B**) Differentiation potential of CD106^+^ and CD106^−^ cells from CV-MSCs. Alizarin red S staining for osteogenic differentiation and oil red O staining for adipogenic differentiation. (**C**) Colony formation ability of CD106^+^ and CD106^−^ cells from CV-MSCs. The number of CFU-F was counted after 14 days culture (*p<0.05).

CV-MSCs possess the strong immunomodulatory potential and express highly CD106. We thus hypothesized that CD106^+^CV-MSCs maybe a novel subpopulation specialized in immunosuppression. To test this hypothesis, the CD106^+^cells and CD106^−^cells were therefore separated from CV-MSCs, either by MACS or SORTING. Both of CD106^+^cells and CD106^−^cells showed typical MSCs morphology (Figure 3A) and osteogenic and adipogenic differentiation abilities (Figure 3B), conforming their identical multipotent stem cell potential. Interestingly, CD106^+^CV-MSCs possessed a five-fold decreased ability for colony formation than CD106^−^CV-MSCs (Figure 3C), suggesting that the CD106 may appear on the MSCs at late developmental stage.

**Figure 4:**
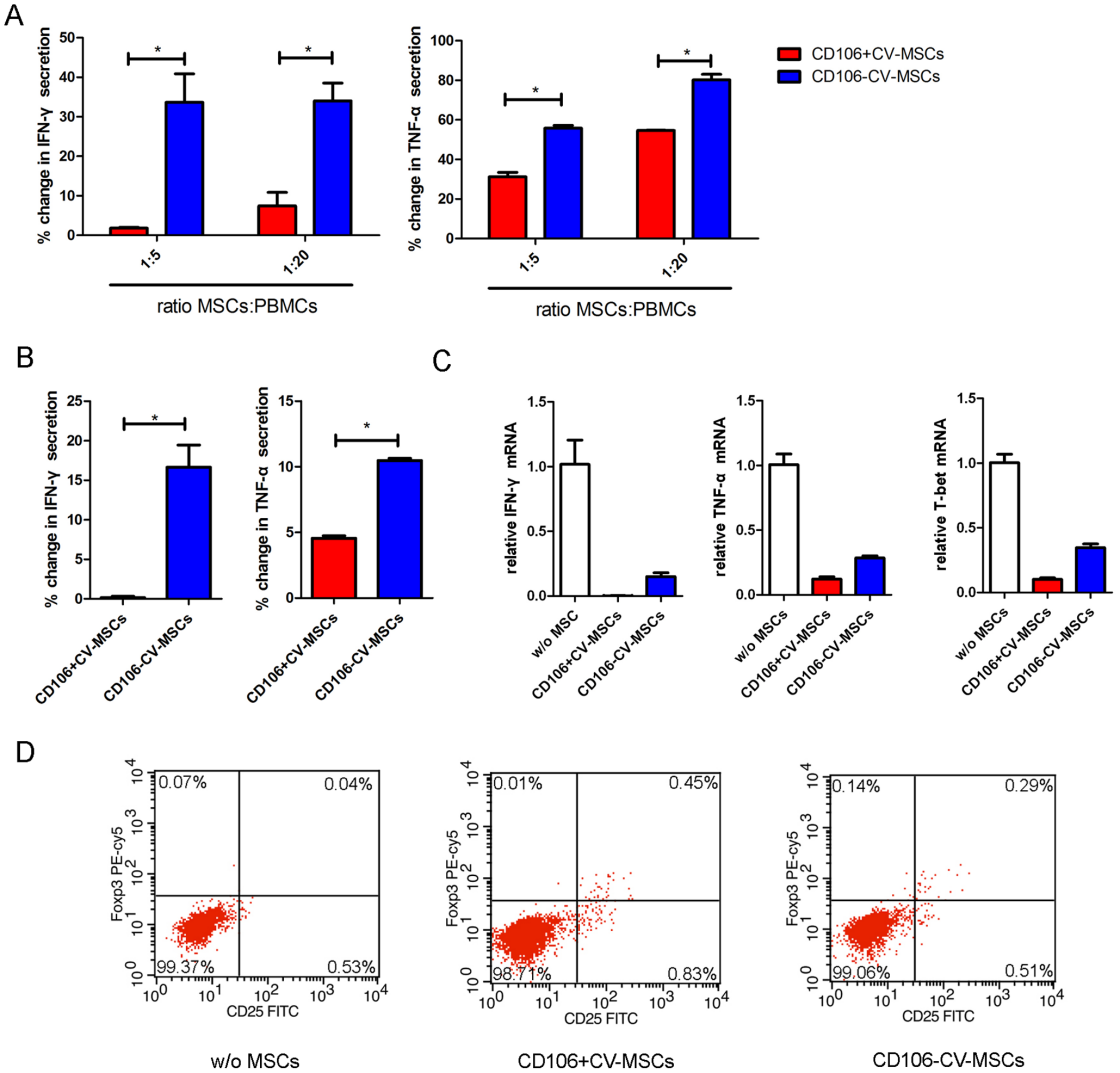
Immune modulation of CD106^+^CV-MSC sand CD106^−^CV-MSCs. (**A**) CD106^+^ or CD106^−^ CV-MSCs were co-cultured with PHA (10 μg/ml) stimulated PBMCs for 72 hours at a ratio of 1∶5 or 1∶20. IFN-γ and TNF-α production of activated-PBMCs were evaluated in co-culture medium by ELISA. Percentage change of IFN-γ and TNF-α was gained by direct comparison to the control (activated PBMCs alone). Data represent the mean of single experiment, each performed in at least triplicate (*p<0.05). (**B**) CD106^+^ or CD106^−^ CV-MSCs were co-cultured with cord blood CD4^+^T cells at a ratio of 1∶10 for 72 hours stimulated with PHA and IL2. IFN-γ and TNF-α production in the supernatant were measured by ELISA. Percentage change of IFN-γ and TNF-α was gained by direct comparison to the control (activated cord blood CD4^+^T cells alone), each performed in at least triplicate (*p<0.05). (**C**) CD106^+^ or CD106^−^ CV-MSCs were co-cultured with cord blood CD4^+^T cells at a ratio of 1∶10 for 72 hours stimulated with PHA and IL2. The relative expressions of IFN-γ, TNF-α and T-bet were measured by realtime PCR. (**D**) CD106^+^ or CD106^−^ CV-MSCs were co-cultured with cord blood CD4^+^T cells at a ratio of 1∶10 for 72 hours stimulated with IL2. In flow cytometric analysis, cells were gated for CD4^+^ cells, and CD25 Foxp3 double positive cells were define as Tregs. Data represent a single experiment, each performed in triplicate.

We then compared the ability of CD106^+^CV-MSCs and CD106^−^CV-MSCs to inhibit the Th1 response by testing the IFN-γ production. CD106^+^CV-MSCs or CD106^−^CV-MSCs did not induce IFN-γ secretion of PBMCs without stimulation. PBMCs secreted IFN-γ upon stimulation of PHA. The addition of CD106^+^CV-MSCs or CD106^−^CV-MSCs resulted in a significant reduction of IFN-γ secretion of PHA stimulated PBMCs, but their inhibitory degree was different. The CD106^+^CV-MSCs induced an almost complete inhibition of IFN-γ secretion whereas CD106^−^CV-MSCs induced only partial inhibition (Figure 4A). In addition, CD106^+^CV-MSCs suppress TNF-α production to a lower level than CD106^−^CV-MSCs (Figure 4A). Furthermore, determination of intracellular cytokine contents on the CD4^+^T cells of the co-cultures showed that both types of CV-MSCs decreased at different degree the expression of IFN-γ and TNF-α, although CD106^+^CV-MSCs were more effective (Figure 4B and C). The expression of T-bet further demonstrated the powerful effect of CD106^+^CV-MSCs on suppressing the Th1 polarization of CD4^+^T cells. In addition, CD106^+^CV-MSCs can induce Tregs more effectively than CD106^−^CV-MSCs (Figure 4D), indicating an important role of CD106^+^CV-MSCs in stimulating the formation of Tregs. In a word, CD106^+^CV-MSCs possess stronger immunosuppressive activity than CD106^−^CV-MSCs.

### CD106^+^CV-MSCs express higher levels of immunoregulatory factors than CD106^−^CV-MSCs

**Figure 5:**
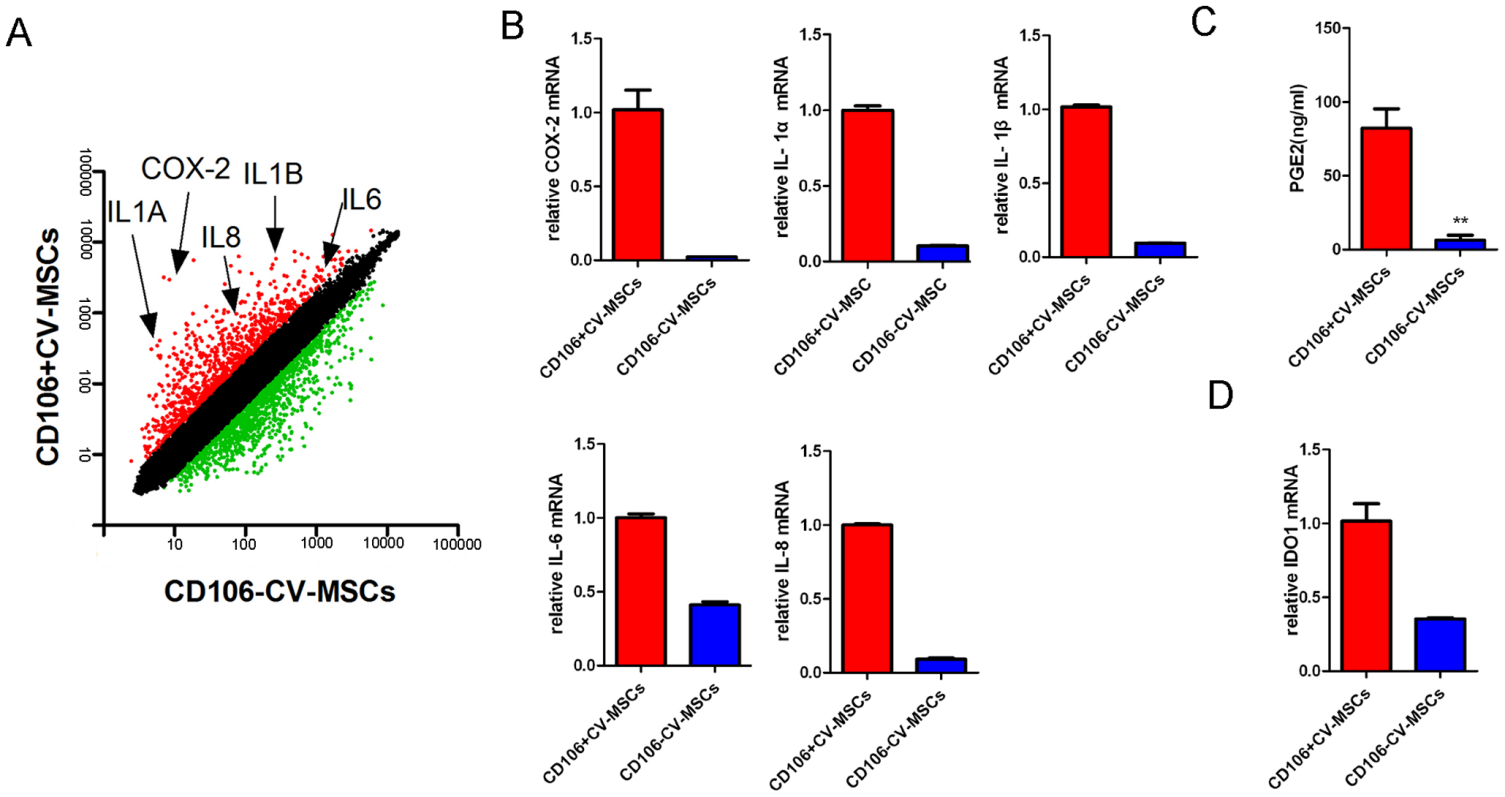
Gene expressions of CD106^+^ and CD106^−^ CV-MSCs. (**A**) The gene expression profile of CD106^+^ and CD106^−^CV-MSCs were determined using Affymetrix oligoarrays and the scatter plot shows the gene expression level. Red plots indicate genes up regulated in CD106^+^CV-MSCs, and green plots indicate genes up regulated in CD106^−^CV-MSCs. (**B**) Relative mRNA expressions of COX-2, IL-1α, IL-1β, IL-6 and IL-8 in CD106^+^ and CD106^−^CV-MSCs were determined by real-time PCR. (**C**) 10^5^ of CD106^+^ or CD106^−^ CV-MSCs were cultured for 72 hours and PGE_2_ concentration in supernatant was measured by ELISA. (**D**) CD106^+^ or CD106^−^ CV-MSCs were cultured with IFN-γ for 24 hours and relative mRNA expressions of IDO1 were determined by real-time PCR. The results are presented as means ± SEM of three separate experiments.

To explore the mechanism underlying increased immunosuppressive function of CD106^+^CV-MSCs, we performed microarray to analyze the gene expression pattern of CD106^+^CV-MSCs and CD106^−^CV-MSCs, and real-time PCR was performed to confirm the results from microarray analysis. The expression of adherence molecules and cytokines were different in CD106^+^ and CD106^−^ CV-MSCs, indicating different functions for two kinds of MSCs. Notably, the gene expression profiles associated with immunoregulatory factors were different, indicating different functions of these two subpopulations (Table 3 and 4). CD106^+^CV-MSCs express higher levels of COX-2, IL-1α, IL-1β, IL-6 and IL-8 compared with CD106^−^CV-MSCs (Figure 5A and B). CD106^+^CV-MSCs showed an increased ability to secrete PGE_2_ compared to CD106^−^CV-MSCs (Figure 5C). To test the ability for another important immune modulator IDO1, IFN-γ was used to stimulate the MSCs. IFN-γ significantly up regulated the expression of IDO1 in 24 hours. CD106^+^cells express higher level of IDO1 than CD106^−^cells in the presence of IFN-γ (Figure 5D). Together, CD106^+^CV-MSCs expressed higher level of immunomodulating factors compared with CD106^−^CV-MSCs.

**Table 3:** Differential pathways of CD106^+^ and CD106^−^ CV-MSCs/.

Pathway	Count	q-Value
Cytokine-cytokine receptor interaction	21	8.40E-15
TGF-beta signaling pathway	11	2.85E-10
Cell adhesion molecules (CAMs)	12	1.88E-09
MAPK signaling pathway	15	8.17E-09
Graft-versus-host disease	5	4.30E-05
Jak-STAT signaling pathway	8	4.62E-05
Chondroitin sulfate biosynthesis	4	5.91E-05
O-Glycan biosynthesis	4	2.11E-04
Focal adhesion	8	2.67E-04
Type I diabetes mellitus	4	6.58E-04
Axon guidance	6	7.15E-04
ECM-receptor interaction	5	7.41E-04
Hematopoietic cell lineage	5	8.33E-04
Wnt signaling pathway	6	0.001391
Glycosphingolipid biosynthesis – lactoseries	3	0.001572
Complement and coagulation cascades	4	0.002474
Melanoma	4	0.002728
Calcium signaling pathway	6	0.002957
Bladder cancer	3	0.00505
Prostate cancer	4	0.005125
ABC transporters – General	3	0.005554
Regulation of actin cytoskeleton	6	0.00583
Gap junction	4	0.00598
Glycerolipid metabolism	3	0.006415
Toll-like receptor signaling pathway	4	0.007696

**Table 4:** Differential biological process associated with CD106^+^ and CD106^−^ CV-MSCs.

GO Term	Count	q-Value
regulation of transcription, DNA-dependent	45	1.26E-37
cell adhesion	29	1.06E-32
development	41	3.25E-32
signal transduction	42	1.13E-26
inflammatory response	19	1.50E-25
pregnancy	13	3.49E-24
positive regulation of transcription from RNA polymerase II promoter	16	2.21E-22
immune response	22	2.88E-21
anti-apoptosis	12	5.55E-17
transcription	27	1.73E-16
cell-cell signaling	16	3.82E-16
negative regulation of cell proliferation	12	6.61E-15
skeletal development	11	1.71E-13
heart development	9	8.75E-13
ion transport	14	1.22E-12
anterior/posterior pattern formation	8	1.40E-12
oxidation reduction	13	2.14E-12
proteolysis	15	1.08E-11
positive regulation of cell proliferation	10	1.08E-11
chemotaxis	8	5.14E-11
negative regulation of transcription from RNA polymerase II promoter	8	1.80E-10
nervous system development	12	9.55E-10
apoptosis	12	1.67E-09
protein amino acid phosphorylation	11	1.67E-09

### CD106 expression on CV-MSCs is controllable in response to propagation and cytokine induction

**Figure 6:**
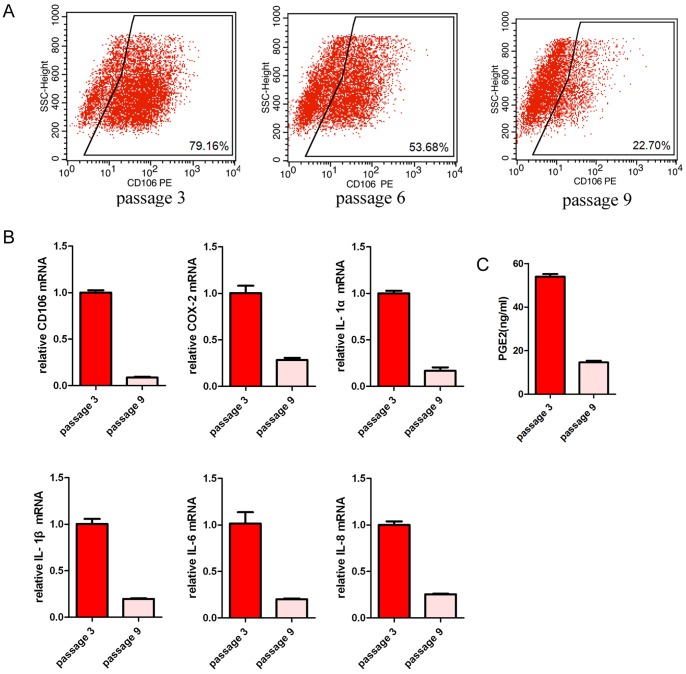
Gene expressions of CV-MSCs changed by passages. (**A**) CD106 expressions in CV-MSCs were detected by flow cytometry. Passages 3, 6, 9 were used. (**B**) Relative mRNA expressions of CD106, COX-2, IL-1α, IL-1β, IL-6 and IL-8 in CV-MSCs were determined by real-time PCR. Passage 3 and passage 9 were used. (**C**) 10^5^ of CV-MSCs were cultured for 72 hours and PGE_2_ concentration in supernatant was measured by ELISA. Passage 3 and passage 9 were used. The results are presented as means ± SEM of three separate experiments.

To determine if the CD106 expression on CV-MSCs is tissue specific and stable during long-term cultivation, we cultured CV-MSCs under different condition. It was interesting to observe that the expression of CD106 decreased after passaging in basic culture media (Figure 6A). Accordingly, there was a reduction of mRNA expression of COX-2, IL-1α, IL-1β, IL-6 and IL-8 (Figure 6B) and a reduction of PGE_2_ production (Figure 6C).

**Figure 7:**
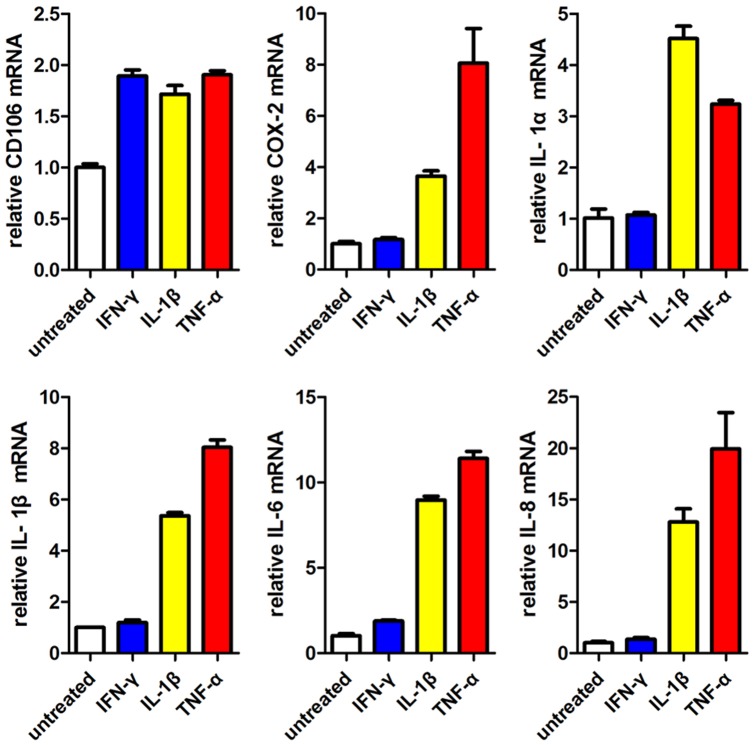
Pro-inflammatory cytokines TNF-α and IL-1β could induce CD106^−^CV-MSCs to up regulate CD106^+^CV-MSCs associate genes. CD106^−^CV-MSCs were cultured with IFN-γ (30 ng/ml), IL-1β (10 ng/ml) or TNF-α (30 ng/ml) for 24 hours respectively. The cells were collected after stimulation, and relative mRNA expressions of CD106, COX-2, IL-1α, IL-1β, IL-6 and IL-8 were measured by real-time PCR. CD106^−^CV-MSCs cultured without pro-inflammatory cytokines were used as the controls. The results are presented as means ± SEM of three separate experiments.

We then wanted to know if CD106^−^CV-MSCs could be induced to express CD106 and thereby increase their immunosuppressive ability. CD106^−^CV-MSCs were treated with IFN-γ, TNF-α or IL-1β for 24 hours. The addition of pro-inflammatory cytokines resulted in an increased expression of COX-2, IL-1α, IL-1β, IL-6 and IL-8 in CD106^−^CV-MSCs (Figure 7). IFN-γ apparently up regulated CD106 and IL-6 expression, but failed in other genes. Together, these results indicate that the expression of CD106 on MSCs is variable, and suggest that TNF-α, IL-1β, but not IFN-γ, may be important for generation of CD106^+^MSCs.

## Discussion

In this study, using CD106 as a surface marker, we have successfully demonstrated a previously uncharacterized heterogeneity of MSCs isolated from chorionic villi. CD106^+^CV-MSCs possess high immunomodulation activity, whereas CD106^−^CV-MSCs possess high colony formation capacity. In addition, we provide several lines of evidence that the immunosuppressive activity of MSCs is highly related to their ability for the secretion of immunoregulatory cytokines and PGE_2_. In CD106^+^cells, a list of genes was up regulated, including COX-2, IL-1α, IL-1β, IL-6 and IL-8. The biological activity of CD106^+^CV-MSCs was highly related to the stimulation of pro-inflammatory cytokines TNF-α and IL-1β.

To our knowledge, CD106 is a cytokine-inducible cell surface protein capable of mediating adhesion to leukocytes expressing alpha 4 integrins. CD106-deficient mouse embryos were not viable and exhibited either of two distinct phenotypes. Half of the embryos died before embryonic day 11.5 and exhibited severe defects in placental development. The remaining CD106-deficient embryos survived to embryonic day 11.5–12.5 and displayed several abnormalities in their hearts development [21]. The role of CD106 in embryonic development is very important for the formation of the umbilical cord and placenta [22].

Human placenta is an important organ to provide nutrition, gas exchange, waste removal, endocrine and immune support during fetus development. The chorionic villi constitute a part of the border between maternal and fetal blood providing exchange between maternal and fetal circulation. Recently, the placenta has been recognized as an immune organ, creating a fetomaternal immune-privileged environment enabling the survival of the semi-allogeneic fetus [23], [24]. The underlying mechanism for the placental immune tolerance has been found to depend on regulatory T cells [25], negative signals from the PD1-PDL1 costimulatory pathway [26], IL-17-producing T cells (Th17) [27] and FAS-Ligand [28]. Th1 immune response should be suppressed, in order to maintain the fetomaternal tolerance. Wegmann and colleagues first developed the concept that there is a shift from a T helper 1 response to a T helper 2 bias during pregnancy that functionally induces maternal tolerance and suppression [29]. Administration of the Th1 interleukin IFN-γ [30] leads to fetal loss and preterm labor in the mouse.

However, little is known about the contribution of MSCs resided in placental chorionic villi to fetomaternal tolerance. We discovered that both of CD106^+^CV-MSCs and CD106^−^CV-MSCs could modulate the IFN-γ secretion, while CD106^+^CV-MSCs were more effective. Surface molecules like CD106 and CD54 are considered to be important for immune modulation of MSCs [19]. Besides, soluble factors are also critical for immunosuppression of MSCs. MSCs modulate the immune response via soluble factors such as PGE_2_ [10], IDO1[31], transforming growth factor beta 1 (TGF-β1) [32], hepatocyte growth factor (HGF) [33], Interleukin 6 (IL-6) [34], HLA-G [35], Nitric oxide (NO) [36], and Galectin-1 [37]. COX-2 is critical for synthesis of inducible PGE2. In CD106^+^cells, COX-2 was highly expressed, which indicated that CD106^+^cells secrete high level of PGE2 mediating the immune modulation function of UC-MSCs [10]. IDO1 can be induced by IFN-γ [31] and CD106^+^CV-MSCs express higher level of IDO1 than CD106^−^CV-MSCs after stimulating with IFN-γ. PGE2 was reported to regulate IDO1 [38], [39]. In our study we also observed that CD106^+^cells are more effective to induce IDO1.

COX-2 expression in fetal membranes seems to be important for pregnancy, as multiple female reproductive failures were observed in COX-2 deficient mice [40]. COX-2 highly expressing CD106^+^cells may have more functions but not only immune modulation. IL-1 is also critical for pregnancy. IL-1Ra prevents embryonic implantation by directly affecting the endometrial epithelium [41]. In addition, human placenta is a potent hematopoietic niche [42], the effect of CD106^+^CV-MSCs on haemopoiesis and angiogenesis may be an interesting field for further study.

Pro-inflammatory cytokines like IFN-γ, IL-1β and TNF-α secreted by immune cells are critical to induce immunomodulatory ability of MSCs [43]–[46]. MSCs stimulated by pro-inflammatory cytokines were shown to be more effective to modulate the immune response in vitro [47] and in vivo [44]. CD106^+^CV-MSCs showed similar gene expressions with CD106^−^CV-MSCs after stimulating with IL-1β or TNF-α, but not IFN-γ. Interestingly, IL-1β and TNF-α have been shown to be important for reproductive [48], [49], while IFN-γ is low at the maternal-fetal interface [29]. To maintain or induce CD106^+^CV-MSCs, stimulus in the microenvironments may be needed, and IL-1β or TNF-α might be candidate stimulus. Certainly other cytokine and cell-cell contact may also participate in the development of CV-MSCs.

In summary, this study reports for the first time that CD106 identifies a unique subpopulation of MSCs with powerful immunosuppressive activity, CD106^+^MSCs mainly reside in human term placenta, which may contribute to fetomatenal tolerance. Our future work will be best directed toward designing therapeutic strategies for the treatment and prevention GVHD or other autoimmnune disorders as well as early pregnancy loss, preterm labour and transplant associated adverse outcomes.
